# Computation Approach Shows Robustness of the Striped Pattern of Fruitfly Embryos

**DOI:** 10.1371/journal.pbio.0020185

**Published:** 2004-06-15

## Abstract

xx

Since the days of ancient Greece, mathematics has been used to describe the world in the hopes of identifying underlying laws of nature. Physicists have long relied on mathematics to understand the behavior and interaction of particles too small to observe directly. Since it's not always possible to determine the behavioral properties of a single atom or electron, physicists characterize the behavior of these particles in terms of probability and the law of averages. Likewise, it's not always easy to tell how a single protein contributes to the behavior of a cell or organism. Faced with increasingly immense datasets—from genomes, proteomes, gene expression networks, cell signaling pathways, and more—biologists are turning to the tools of higher mathematics. High-throughput technologies like genome sequencers and microarrays generate a global picture of genomic or cellular activity, but such datasets have a high noise-to-signal ratio—the details are often subject to multiple interpretations. One way computational methods can help separate the signal from the noise is by determining the likelihood of a given set of interactions and presenting a range of possible network behaviors. When sufficient information about a biological pathway is available, experimental evidence can enhance modeling approaches to help refine the nature and role of putative network behaviors. (To learn more about computational biology, see the essay “A Calculus of Purpose,” by Arthur Lander, also in this issue of *PLoS Biology*.)[Fig pbio-0020185-g001]

**Figure pbio-0020185-g001:**
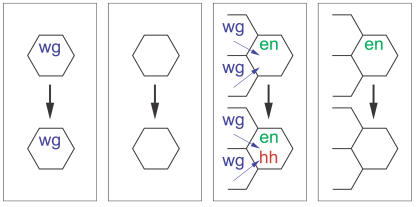
Cell behaviors for segment polarity patterning

One model system ripe for computational analysis is the fruitfly Drosophila melanogaster, genetically the best-understood multicellular organism. Drosophila development proceeds through a complex series of both sequential and simultaneous events. An elaborate network of genetic interactions transforms a single-cell Drosophila egg into a multicellular embryo with 14 discrete segments. These segments are the result of a series of hierarchical decisions, as one set of genes induces the characteristic expression pattern of another set: “gap” genes direct the striped expression pattern of “pair rule” genes, which induce expression of segment polarity genes, whose messenger RNA (mRNA) and protein products produce the characteristic 14-segment polarity pattern. While the molecules and pathways that generate the segment polarity pattern are well known, little is known about the quantitative nature of their interactions: in what concentrations do the components (for example, mRNAs and proteins) exist and what parameters (for example, binding constants, transcription rates, and gene product life spans) govern their interactions?

Four years ago a group of researchers led by George von Dassow developed a model of the genetic interactions that define segment polarity, called the segment polarity network. The model used a parameter set of 48 numerical values for each computer simulation of the segment polarity pattern. Since quantitative information about the network was unavailable, the group used random values for each of the parameters, repeating the simulation for nearly 250,000 different random parameter sets. The model proved remarkably robust—the network output was largely insensitive to variation in parameter values, with a surprisingly large fraction of random parameter sets generating the desired segment polarity pattern. That so many random variables could produce the pattern means either that almost any set of parameter values can work or that only a few of the parameters are important. Now, Nicholas Ingolia reveals the mechanism accounting for this robustness and bolsters the model with recent experimental evidence.

To investigate the reason for the original model's robustness, Ingolia asked whether the parameters of the model could be deconstructed into the properties of individual cells. It's known, for example, that the stable expression of two genes, called *wingless (wg)* and *engrailed (en)*, within specific cells of a “prepattern” laid down early in embryogenesis is converted into the segment polarity pattern by an intercellular signaling network. *Wg* and *en* operate through positive feedback loops that activate their own expression, a process that is destined to end up with individual cells in one of two stable states of gene expression (an outcome called bistability). Since each stable state is intrinsically robust—that is, resistant to changing parameters—Ingolia hypothesized that the parameters that generate the robustness of the segment polarity pattern in von Dassow's model are those that produce this bistability.

Using computational methods to simulate the behavior of individual cells, Ingolia shows that individual cells in the original model adopt three different stable states of *wg* and *en* expression. The overall pattern of the model, as well as its insensitivity to parameter variation, Ingolia concludes, emerges from the stable expression states of single cells. Parameters that do not produce bistability within single cells, Ingolia found, almost never generate the correct pattern, while those that do produce bistability are much more likely than randomly chosen parameters to generate the striped segment pattern. When Ingolia added new experimental variables to the model—the signaling protein produced by the *sloppy-paired* gene and its interactions with *en*—he could reduce the fraction of parameter sets that satisfied the bistability requirement but nonetheless failed to produce the segment polarity pattern, refining the model to reflect the realities of the cell.

Such computational approaches are allowing biologists to gain valuable insights into the real-world properties and behavior of staggeringly complex biological networks. It's been over 2,000 years since Pythagoras proposed that the laws of heaven and earth reflect a numerical harmony rooted in mathematical laws. Whether that notion holds for biology, bit by bit the tools of higher mathematics are peeling back the layers of complexity to identify underlying properties of living systems.

